# Supplementary material for: Augmented Global Protein Acetylation Diminishes Cell Growth and Migration of Cholangiocarcinoma Cells
Source: Int J Mol Sci. 2024 Sep 22;25(18):10170. doi: 10.3390/ijms251810170 (PMC11432552; doi:10.3390/ijms251810170)
Supplement: Supplementary file 1 [file ijms-25-10170-s001.zip › ijms-3197487-supplementary.pdf]

Supplementary Figure S1

(a)

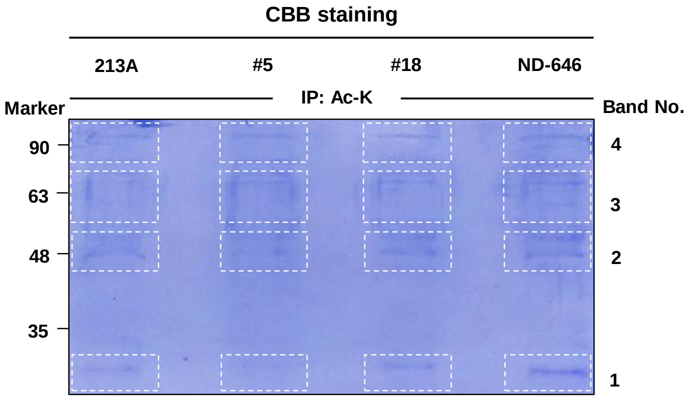

(b)

Band	213A	#5	#18	ND-646
No.1	H12<br>H15	H12<br>SOX3<br>TACO1	H12<br>H15	H12<br>H15
No.2	AN34B<br>EF1A1<br>PO6F2<br>TBA1C<br>TBB4A<br>TBB4B<br>TBB5	ACTB<br>ACTBL<br>EF1A1<br>K2C1<br>PCDC1<br>POTEE<br>RB3GP<br>TBA1B<br>TBB4A<br>TBB5<br>YBOX1	ACTB<br>ACTBL<br>CSMD2<br>DNHD1<br>EF1A1<br>POTEE<br>TBA1B<br>TBB4B<br>TBB5B<br>TBB8B	EF1A1<br>POC1B<br>POTEE<br>RL4<br>TBA1A<br>TBB4A<br>TBB4B
No.3	ASAP2<br>HS71A<br>HSP7C	ASAP2<br>EVG1<br>PEX1		PEX1
No.4	TACO1	HSP90B	HSP90A<br>HSP90B<br>PEX1<br>TRAP1	HSP90B<br>PEX1<br>TRAP1

(c)

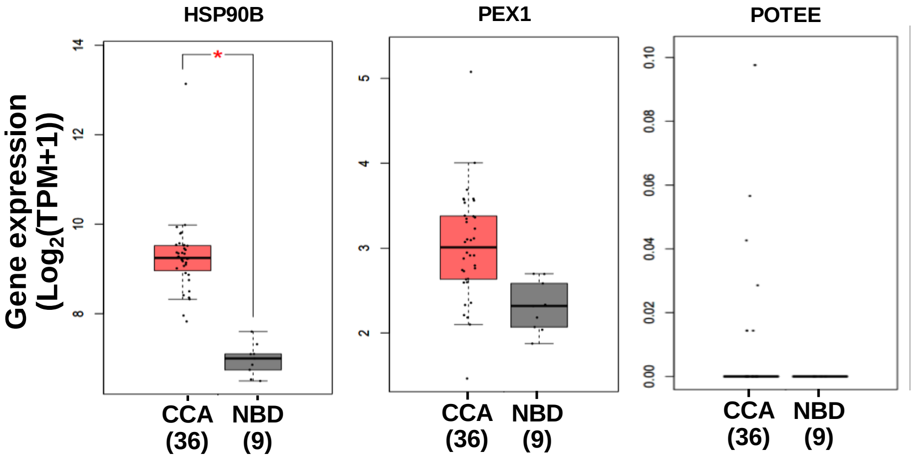

Figure S1. Acetylated proteins in control (213A) and three ACC1 inhibitory conditions (ACC1-KD clones#5, #18, and 0.5 µM ND-646-treated cells) were identified, and the clinical significance of HSP90 is highlighted by preliminary analysis using public database GEPIA. (A) Immunoprecipitated acetylated proteins were prepared from four conditions: KKU-213A (213A) parental cells, two ACC1-KD clones (#5 and #18), and ND-646 treated cells, and subjected to SDS-PAGE and Coomassie blue staining. The white dashed boxes represent the interested bands, which were cut and subjected to in-gel tryptic digestion and tandem mass spectrometry analysis (LC-ESI-MS/MS). (B) Identified proteins from each polyacrylamide fragment were listed. Three common proteins identified in all ACC1 inhibitory conditions are highlighted in blue. (C) The box plots represent HSP90B, PEX1, and POTEE mRNA expression of human CCA tissues (n=36) compared to normal bile duct (NBD) (n=9) retrieved from GEPIA database. TPM; transcript per million.

Supplementary Figure S2

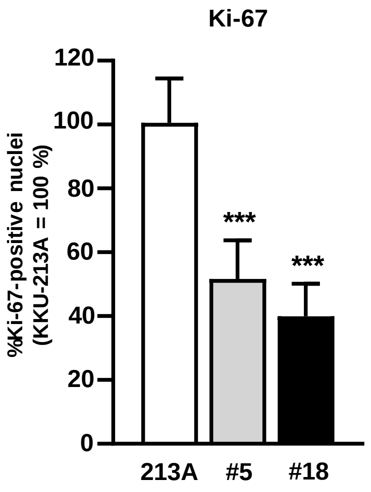

Figure S2. The effects of ACC1 deficiency on Ki-67 expression *in vivo*. The numbers of Ki-67-positive nuclei in Figure 7e were counted (n=5/group, 20X objective lens). Ki-67-positive nuclei of KKU-213A-injected group = 100%. 213A; KKU-213A-injected group, #5; 13AC1-KD#5-injected; #18; 13AC1-KD#18-injected, CK-19; ACC1-KD clones cytokeratin 19, Ki-67; Ki-67 antigen. ***$p<0.001$.

Supplementary Figure S3

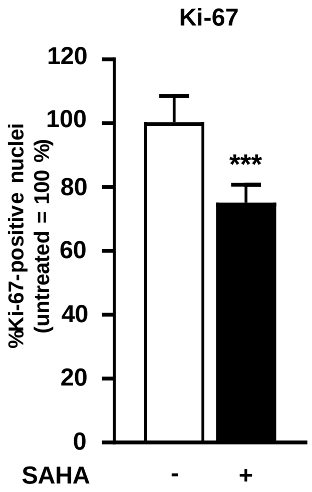

Figure S3. The effects of SAHA treatment on Ki-67 expression in CCA xenografted mouse model. The numbers of Ki-67-positive nuclei in Figure 8e were quantitated (n=5/group, 20X objective lens). Ki-67-positive nuclei of untreated group = 100%. The CK-19; cytokeratin 19, Ki-67; Ki-67 antigen. ***p<0.001.
